# Warfarin-Induced Skin Necrosis in a 14-Year-Old Female: A Case Report

**DOI:** 10.7759/cureus.30354

**Published:** 2022-10-16

**Authors:** Tafseer Zahra, Sidra Jamil, Hayder Ferman, Yuvapriya Ravikumar, Diana Voloshyna, Tanveer Ahamad Shaik, Faraz Saleem, Muhammad Abu Zar Ghaffari

**Affiliations:** 1 Medicine, California Institute of Behavioral Neurosciences and Psychology, Fairfield, USA; 2 Internal Medicine, Dr. Mary Gallagher Clinic, Virginia, USA; 3 Internal Medicine, State University of New York Downstate Health Sciences University, New York, USA; 4 Internal Medicine, University of Michigan, Ann Arbor, USA; 5 Cardiovascular Medicine, University of Louisville School of Medicine, Louisville, USA; 6 Internal Medicine, Akhtar Saeed Medical and Dental College, Lahore, PAK

**Keywords:** drug toxicity, necrosis, warfarin toxicity, warfarin-induced skin necrosis, warfarin

## Abstract

Warfarin is a widely known oral anticoagulant used for the treatment and prevention of thromboembolic conditions. A rare, crippling, and occasionally fatal complication of warfarin is skin necrosis, resulting in significant morbidity and mortality. Due to the disease's unknown pathophysiology and rare occurrence, the treatment guidelines are not well established. We present the case of a 14-year-old female with a history of mitral stenosis and atrial fibrillation who had been on warfarin for the last two years and now develops an acute excruciating rash within three days of reinitiation of warfarin despite enoxaparin bridging and a normal blood clotting profile. After cessation of warfarin, the skin necrosis progressed to eschar formation and resolved within four weeks. To prevent further complications, early diagnosis and treatment with intravenous vitamin K, fresh frozen plasma (FFP), and aggressive wound care are essential. The prognosis may be improved by prompt diagnosis and drug cessation.

## Introduction

Warfarin is an oral anticoagulant that is commonly prescribed to patients with venous thromboembolism, pulmonary embolism, and mechanical heart valve replacements. Several common adverse effects, such as bleeding and skin necrosis, have been linked to the use of warfarin [1]. There is a rare but devastating side effect of oral anticoagulant therapy termed warfarin-induced skin necrosis (WISN) [2]. It typically occurs within the first few days of starting warfarin [3].

The use of warfarin without bridging therapy appears to be linked to this adverse effect, especially when a large loading dose of the drug is introduced [3]. Significant morbidity from WISN can be avoided through early diagnosis and treatment [3]. We present the case of a child who presented with an excruciating erythematous rash on the back, which was later diagnosed with WISN.

## Case presentation

A 14-year-old female patient arrived at the hospital's emergency room complaining of the sudden onset of a tender erythematous rash on her back. Two years ago, she presented to the hospital's emergency department with an embolic stroke and was later diagnosed as having mitral stenosis with atrial fibrillation. Since then, she has been on 5 mg of warfarin daily and has maintained a therapeutic international normalized ratio (INR) of 3.

Until recently, when she had an upper respiratory bacterial infection and was self-prescribed ceftriaxone as an antibiotic of choice by her parents, her INR spiked to 9.2 within a week of taking the antibiotic. Warfarin was immediately stopped, and 1.0 mg of intravenous vitamin K was administered to the patient. She was admitted to the hospital to observe her condition further. Her INR level returned to 2.4 in less than a day. After beginning treatment with enoxaparin for three days, the patient was switched to her usual dose of 5 mg of warfarin, and the enoxaparin was stopped. The patient was discharged on an INR level of 2-3, and her family was counseled against self-medication.

**Figure 1:**
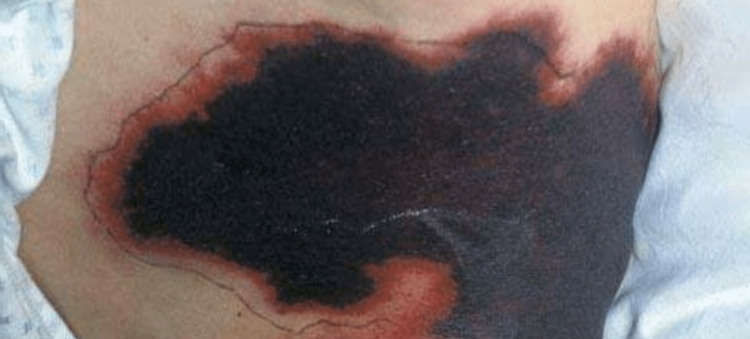
Warfarin-induced skin necrosis

However, within three days of being discharged, the patient returned to the hospital's emergency department with painful back lesions. Examining her back revealed skin lesions with coalescing ecchymoses and incredibly tender erythematous lesions that were extremely painful to the touch (Figure 1).

There were also some areas of necrosis. Numerous laboratory tests were conducted including protein-c, protein-s, INR, and the clotting factor assay. All were discovered to be within normal limits (Table 1).

**Table 1:** Lab parameters MCV: Mean corpuscular volume; AST: Aspartate transaminase; ALT: Alanine aminotransferase; BUN: Blood urea nitrogen; aPTT: Activated partial thromboplastin time; PT: Prothrombin time; INR: International normalized ratio.

Complete blood count parameters	Values
Hb (g/dL)	12.6 (13.5–17.5)
MCV (fl)	78.8 (80–100)
WBC (x 10^9^/L)	5.2 (4.5–11)
Platelets (x 10^3^/uL)	360 (150–400)
ALT (IU/L)	24 (7–55)
AST (IU/L)	33 (8–48)
BUN (mg/dL)	24 (6–24)
Cr (mg/dL)	1.1 (0.7–1.3)
aPTT (seconds)	33 (21–35)
PT (seconds)	19 (11–13.5)
INR	3 (1.1 or below)
Protein-C (IU/dL)	102 (65–135)
Protein-S (U/dL)	90 (60–130)

After carefully examining and reviewing her medical history, she was diagnosed with WISN. The patient was admitted immediately, and her warfarin was replaced with enoxaparin, vitamin K, and FFP. Her INR was three at admission.

During the two-week course of therapeutic enoxaparin, the patient's lesions stabilized and began to regress. Within four weeks, she was healed, and her lesions also receded. Given her history of stroke and mitral stenosis, she was once more started on 2.5 mg of warfarin with enoxaparin for the next 11 days, then gradually increased her warfarin to a final dose of 5 mg, and enoxaparin was discontinued. Her INR levels were monitored regularly during her stay at the hospital and were found to be in the therapeutic range. She was discharged one month after her initial presentation and was followed up regularly for the next three months. The patient fully recovered, and her family received information and guidance regarding the patient's condition.

## Discussion

Around 300 cases of warfarin-related necrosis have been reported worldwide [1]. Most cases of warfarin-induced skin lesions appear within the first few days of treatment, but others have been reported up to three years later [4]. Even though most cases manifest within one to 10 days, some have been reported to manifest as late as three years after the treatment began [5]. Breasts, abdomen, buttocks, calves, and thighs are more prone to necrosis [4] because of their increased subcutaneous fat, likely due to the poor blood supply to adipose tissue [6]. Paresthesia, a feeling of pressure, and a red, flushed face are prodromal features [7]. Eventually, the underlying skin necrosis leads to blistering and the development of large ecchymotic patches, or petechiae [1]. Increased mortality is associated with the rapid development of skin necrosis from plaques. When warfarin therapy is started with a high-loading dose or without concomitant heparin, skin necrosis often develops rapidly [6]. Full-thickness skin loss and necrotic eschar develop after infarction [4]. WISN can be distinguished from disseminated intravascular coagulation (DIC), heparin-induced skin necrosis, and necrotizing fasciitis based on clinical history, cutaneous distribution, laboratory investigations, and histological features, as mentioned previously [8].

Warfarin's anticoagulant effect results from its inhibition of vitamin K-dependent gamma-carboxylation of clotting factors II, VII, IX, and X. On the other hand, natural anticoagulant protein-C and protein-S would also get affected by the same process [9]. Considering that the concentration of anticoagulant protein-C declines more rapidly than other vitamin K-dependent procoagulant factors, which have longer half-lives [10-12], this may lead to a paradoxical hypercoagulable environment wherein microthrombi develop in cutaneous and subcutaneous venules. Hypersensitivity reaction, deficiency in protein-C, and a direct toxic effect of warfarin are all possible common etiological mechanisms [4,8]. WISN is not pathognomonic for protein-C deficiency; it has been described in individuals with other inherited thrombophilia, such as factor V Leiden mutation and protein-S deficiency, and transient reductions of protein-C levels (e.g., in the setting of cancer) [13]. However, our patient had a therapeutic range of INR at the time of discharge. She also had no family history of protein-C deficiency or purpura fulminans.

Unfortunately, the root cause of WISN has not been well established in the literature; however, microvascular thrombosis has previously been considered the leading cause of skin necrosis [14]. It is possible that multiple factors contribute to the pathology, and the evidence for the dominant hypotheses is scant [1]. Hematoma, purpura fulminans, DIC, cellulitis, necrotizing fasciitis, calciphylaxis, and venous gangrene were all considered possible causes [6,10]. The diagnostic tests for protein-C and protein-S concentration are neither sensitive nor specific. Although a number of assays are available, none of the available assays will be accurate during warfarin therapy, and some clotting-based assays may also be affected by other anticoagulants. So, a clinical diagnosis and consultation with the testing laboratory may be advisable [6,13].

All patients, even those with an initially normal clotting profile, should be monitored closely for signs of skin necrosis during aggressive warfarinization. The prognosis is greatly improved by a rapid diagnosis and the subsequent stopping of warfarin [6].

Warfarin is known to induce skin necrosis in extremely rare cases, but prescribers should be aware of this potential risk. The likelihood of developing this potentially fatal condition may be reduced with effective bridge therapy [3]. Withdrawal of the offending drug is the cornerstone of treatment for adverse drug events [1]. Anticoagulation should be continued with a parenteral agent once it is deemed appropriate. The treatment consists of discontinuing warfarin and reversing the drug's effect on protein-C with vitamin K, fresh frozen plasma (FFP), and prothrombin complex concentrate (PCC), in addition to providing wound care [3,4,13]. After discontinuing warfarin, a therapeutic dose of heparin, enoxaparin, in this case, is started. In severe cases of protein-C deficiency, protein-C concentrates may be administered [8]. Patients may need skin grafting or local debridement [4]. Deep tissue necrosis, sepsis syndrome due to wound infection, and multiorgan failure are the leading causes of death [15].

For patients who have experienced WISN and need long-term anticoagulation, there are currently no clear guidelines for reintroducing warfarin. It has been proposed that a gradual achievement of the therapeutic dose of warfarin may be an effective strategy for preventing this condition and can substantially reduce the risk of developing WISN. The transient procoagulant effect of warfarin can be countered by anticoagulation with heparin, so it is advised to increase the dose of both drugs gradually until the target INR is reached [16].

## Conclusions

WISN, although not common and particularly rare in chronic users, should be suspected in any patient receiving warfarin therapy. If not promptly investigated and treated, it can result in serious consequences. Patients who receive early treatment have an excellent prognosis and can continue to use warfarin later.
